# Supplementary material for: Deletion of wfs1 Impairs Oligodendrocyte Precursor Cells Dorsal Distribution and Myelination Through the wfs1-hmgcs1 Axis in Zebrafish
Source: Biology (Basel). 2026 Aug 16;15(16):1402. doi: 10.3390/biology15161402 (PMC13510151; doi:10.3390/biology15161402)
Supplement: Supplementary file 1 [file biology-15-01402-s001.zip › File S1-Transcriptome sequencing data and differentially expressed genes (DEGs) in wfs1-- zebrafish at 5 dpf/2.KEGG/wfs1KOvsWT/DOWN/src/dre03040.html]

dre03040


- dre:286774

- Down regulated genes

286774\_1(-1.42217795837045)

- dre:100037380

- Down regulated genes

100037380\_2(-7.188582372824)

- dre:406288

- Down regulated genes

406288\_1(-1.12731177210543)

- dre:100004402

- Down regulated genes

100004402(-1.74003077548578)

- dre:30671

- Down regulated genes

560210(-2.35591332225681)

- dre:566017

- Down regulated genes

566017(-1.85661913518901)

- dre:100005893

- Down regulated genes

100005893\_1(-1.31383589537958)

- dre:394058

- Down regulated genes

394058\_1(-1.99218425808186)

Close
